# Uncertain-tree: discriminating among competing approaches to the phylogenetic analysis of phenotype data

**DOI:** 10.1098/rspb.2016.2290

**Published:** 2017-01-11

**Authors:** Mark N. Puttick, Joseph E. O'Reilly, Alastair R. Tanner, James F. Fleming, James Clark, Lucy Holloway, Jesus Lozano-Fernandez, Luke A. Parry, James E. Tarver, Davide Pisani, Philip C. J. Donoghue

**Affiliations:** 1 School of Earth Sciences, University of Bristol, Life Sciences Building, 24 Tyndall Avenue, Bristol BS8 1TQ, UK; 2 School of Biological Sciences, University of Bristol, Life Sciences Building, 24 Tyndall Avenue, Bristol BS8 1TQ, UK; 3 Department of Life Sciences, Natural History Museum, Cromwell Road, London SW7 5BD, UK

**Keywords:** phylogeny, Bayesian, parsimony, cladistics, morphology, palaeontology

## Abstract

Morphological data provide the only means of classifying the majority of life's history, but the choice between competing phylogenetic methods for the analysis of morphology is unclear. Traditionally, parsimony methods have been favoured but recent studies have shown that these approaches are less accurate than the Bayesian implementation of the Mk model. Here we expand on these findings in several ways: we assess the impact of tree shape and maximum-likelihood estimation using the Mk model, as well as analysing data composed of both binary and multistate characters. We find that all methods struggle to correctly resolve deep clades within asymmetric trees, and when analysing small character matrices. The Bayesian Mk model is the most accurate method for estimating topology, but with lower resolution than other methods. Equal weights parsimony is more accurate than implied weights parsimony, and maximum-likelihood estimation using the Mk model is the least accurate method. We conclude that the Bayesian implementation of the Mk model should be the default method for phylogenetic estimation from phenotype datasets, and we explore the implications of our simulations in reanalysing several empirical morphological character matrices. A consequence of our finding is that high levels of resolution or the ability to classify species or groups with much confidence should not be expected when using small datasets. It is now necessary to depart from the traditional parsimony paradigms of constructing character matrices, towards datasets constructed explicitly for Bayesian methods.

## 1. Introduction

The fossil record affords the only direct insight into evolutionary history of life on the Earth, but the incomplete preservation and temporal distribution of fossils has long prompted biologists to seek alternative perspectives, such as molecular phylogenies of living species, eschewing palaeontological evidence altogether [1]. However, there is increasing acceptance that analyses of historical diversity cannot be made without phylogenies that incorporate fossil species [2,3] and calibrating molecular phylogenies to time cannot be achieved effectively without recourse to the fossil record [4]. Integrating fossil and living species has become the grand challenge and there has been a modest proliferation of phylogenetic approaches to the analysis of phenotypic data. While conventional parsimony remains the most widely employed method, alternative parsimony [5] and probabilistic [6] models have been developed to better accommodate heterogeneity in the rate of evolution among characters and across phylogeny. Unfortunately, these competing methods invariably yield disparate phylogenetic hypotheses among which it is difficult to discriminate as the true tree is never known for empirical data.

A number of studies have attempted to establish the efficacy of competing phylogenetic methods using data simulated from known trees [7–9], finding that the probabilistic Mkv model outperforms parsimony methods, among which, conventional equal-weights parsimony (EW-Parsimony) performs best. However, these studies were potentially biased by their experimental design: (i) two of the studies employed a generating tree that was unresolved and, therefore, biased against parsimony methods which recover resolved trees; (ii) these studies did not discriminate between the impact of the probabilistic model and its implementation in a Bayesian framework; (iii) based on single empirical trees, the impact of tree symmetry, which is known to confound phylogeny estimation [10], was not explored; and (iv) only binary characters were considered, whereas empirical datasets are commonly a mixture of binary and multistate characters. Therefore, we compare the performance of EW-Parsimony, implied-weights parsimony (IW-Parsimony), maximum-likelihood and Bayesian implementations of the Mk model, based on datasets with different numbers of characters, comprising binary and multistate characters and simulated on a fully balanced and a maximally imbalanced phylogenetic tree. We find that Bayesian inference outperforms all other methods, while EW-Parsimony performs better than IW-Parsimony, and maximum likelihood performs worst of all. We apply these competing phylogenetic methods to empirical morphological datasets of similar sizes to our simulated datasets and explore the efficacy of the ensuing phylogenetic hypotheses in the light of the conclusions derived from our simulation-based study.

## 2. Material and methods

### (a) Simulation of morphological matrices

We simulated data on two 32-taxon generating trees at the extremes of tree symmetry: one fully asymmetrical and one fully symmetrical (see electronic supplementary material, figure S1). For each tree, we simulated matrices of three sizes: 100, 350 and 1000 characters. We generated matrices using the HKY + Γ Continuous model of molecular substitution, with *κ* = 2, the shape (set equal to rate) of the gamma distribution and underlying substitution rate for each replicate sampled from independent and identically distributed exponential distributions with a mean of 1, and character state stationary frequencies fixed as *π* = [0.2,0.2,0.3,0.3]. We used a fixed and uneven stationary distribution of nucleotide frequencies to ensure our simulation model did not collapse into the Mk model, as this would bias the analysis in favour of Mk model-based approaches. We simulated 1000 replicate matrices with unique substitution parameters for each tree and each character number, resulting in a total of 6000 matrices. We set two types of character within each matrix, binary and multistate, and we simulated a proportion of 55 binary : 45 multistate characters, based on the mean ratio found in a survey of empirical morphological data matrices [11]. We established binary characters by converting data simulated under the HKY model to R/Y coding (i.e. 0/1): morphological multistate characters were simulated by converting DNA bases to integers.

To ensure that our simulated data are realistic, we generated each set of 1000 unique replicate matrices such that the among-matrix distribution of homoplasy approximated the distribution of empirical homoplasy, characterized by the consistency index (CI), reported by Sanderson & Donoghue [12]. To approximate this distribution of homoplasy, we placed the Sanderson and Donoghue data into quantized bins of CI spanning 0.05, between the empirical bounds of 0.26 and 1.0, and simulated matrices until we matched this expected density per bin (electronic supplementary material, figure S2).

The code used to simulate these data is available in the electronic supplementary material.

### (b) Phylogenetic analysis

We analysed the simulated matrices with EW-Parsimony, IW-Parsimony (*k* = 2) and the Mk model [6] under both maximum-likelihood and Bayesian implementations. EW-Parsimony and IW-Parsimony estimation of topology was performed in TNT [13]. We used the Mk + Γ model for maximum-likelihood estimation of topology in RAxML v. 7.2 [14], and Bayesian estimation of topology in MrBayes v. 3.2 [15]. As the approximate likelihood calculation of RAxML may be distant from the true likelihood [16], we conducted a sensitivity test by re-analysing a subset of our data with the likelihood implementation of the Mk model in IQ-tree [17]; both methods gave effectively identical results, indicating results from the likelihood Mkv model are not software specific.

The Mkv model is inappropriate due to the lack of acquisition bias in the simulated data. For maximum-likelihood and Bayesian analyses, we applied the discretized gamma distribution model to account for between-character rate heterogeneity. For Bayesian analyses, the posterior distribution was sampled 1 million times by four chains using the Metropolis-coupled Markov-chain Monte Carlo algorithm with every 100th sample stored, resulting in 10 000 samples; two independent runs were performed for each replicate and the two resulting posterior samples were combined after qualitative assessment of convergence. For parity, we characterized the result of all phylogenetic methods as the majority-rule consensus of resultant tree samples. We did not employ bootstrap methods to measure support for parsimony and likelihood analyses because phenotypic data does not meet the assumption that phylogenetic signal is distributed randomly among characters.

We used the Robinson–Foulds metric [18] to compare the similarity of estimated topologies against their respective generating tree. We also noted the per-node resolution, and the variation of node accuracy across the topology.

### (c) Empirical analyses

We analysed four published palaeontological phenotype character matrices that encompass a range of character numbers and a diverse sample of taxa from the Tree of Life [19–22]. We resolved any ambiguities in character coding to their most derived state for each matrix to make analyses compatible across the different phylogenetic methods, facilitating comparison of results. We analysed each matrix by applying the same settings used to analyse our simulated matrices: EW-Parsimony, IW-Parsimony, as well as Bayesian and maximum-likelihood implementations of the Mk model. Empirical morphological matrices are rarely constructed to contain invariant or parsimony uninformative characters. Therefore, the Mkv extension of the Mk model, which uses conditional likelihood to correct for such acquisition biases, is more appropriate than the Mk model for analysis of these empirical data matrices [6].

## 3. Results

### (a) Simulated data

**Figure 1.**
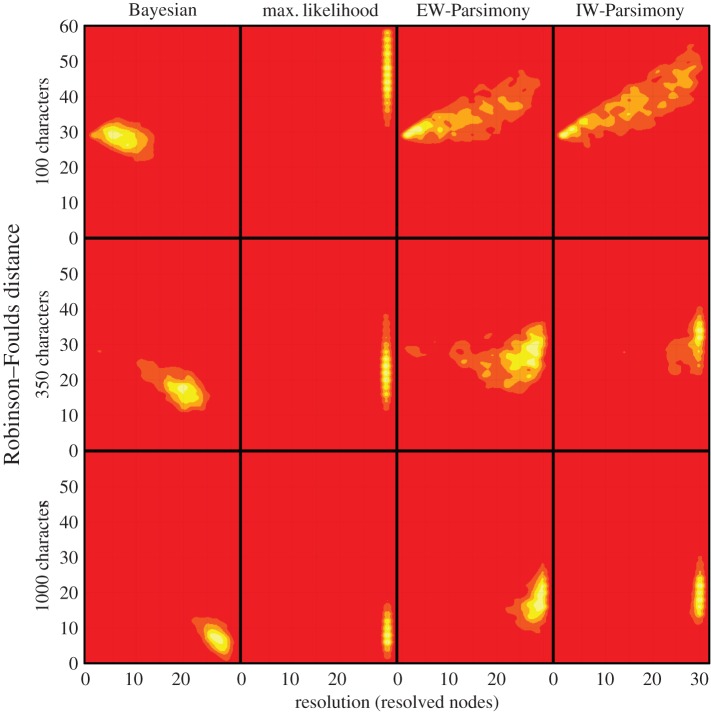
Contour plots of Robinson–Foulds distance against phylogenetic resolution, indicating the higher accuracy of Bayesian implementations against all other methods with data generated on the asymmetrical phylogeny. The spectrum of red to yellow, reflect lower to higher density of trees. As the number of characters increases all methods converge on the correct phylogeny, although Bayesian phylogenies are generally the least resolved. The other methods achieve higher resolution but at a cost of lower accuracy. Data generated on the symmetrical phylogeny shows similar patterns but with much less variance and higher accuracy for all iterations; this lack of variance means point estimates cannot be shown as density estimates. (Online version in colour.)

**Figure 2.**
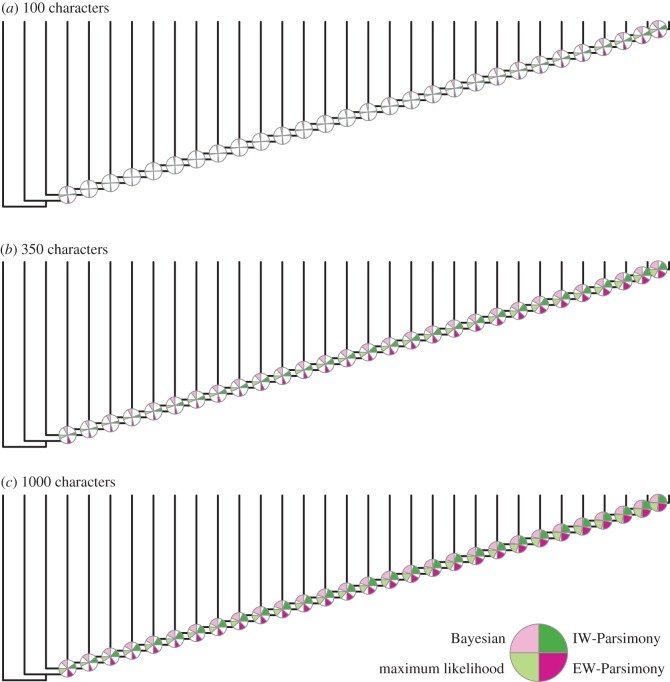
Accuracy of nodes is higher for those closer to the tips in the asymmetrical trees. The percentage of times a node was accurately reconstructed is shown as a proportion of a quarter of a circle in anticlockwise order for Bayesian, maximum likelihood, EW-Parsimony and IW-Parsimony at each node. Accuracy of reconstructions is significantly lower in the 100 character dataset (*a*), and increases in the 350 character (*b*) and 1000 character datasets (*c*). (Online version in colour.)

**Figure 3.**
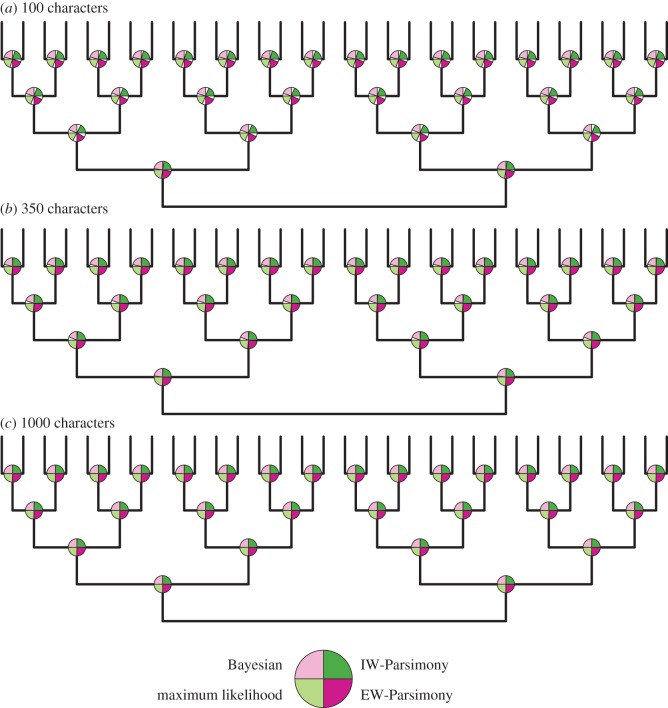
Accuracy of nodes is high for all nodes in the symmetrical phylogeny. The percentage of times a node was accurately reconstructed is shown as a proportion of a quarter of a circle in anticlockwise order for Bayesian, maximum likelihood, EW-Parsimony and IW-Parsimony at each node. Accuracy of reconstructions is high in each dataset size, but there is a non-significant increase in accuracy as dataset size increases (*a–c*). (Online version in colour.)

Accuracy is higher for trees inferred from data simulated on a symmetrical topology compared with trees estimated from data simulated on the asymmetrical topology (cf. figures 2 and 3). Bayesian consensus phylogenies are generally the least well-resolved (figure 1). All methods estimated topologies with greater accuracy as the number of analysed characters increased (figures 2 and 3; electronic supplementary material, table S5–S7). All methods, apart from maximum likelihood, produced phylogenies with greater resolution with higher numbers of characters (figure 1).

For all implementations and dataset sizes, the Bayesian implementation of the Mk model achieves higher accuracy compared with other methods (table 1; figures 1–3). The two parsimony methods achieved the next highest levels of accuracy, EW-Parsimony achieving greater accuracy than IW-Parsimony. Maximum likelihood was the least accurate method for topology reconstruction for both the symmetrical and asymmetrical phylogenies (table 1). The relative accuracy of these phylogenetic methods remains the same across all dataset sizes and the two simulation topologies (table 1; figures 1–3).

**Table 1.** Bayesian approaches produce the most accurate trees for all character sets. Mean and range (in brackets) of Robinson–Foulds distances are lower for topologies estimated using Bayesian methods for both the symmetrical and asymmetrical generating tree. Maximum likelihood is the generally the most inaccurate method for the symmetrical generating tree, and implied weights parsimony performs worst for the asymmetrical generating tree.

	equal weights parsimony	implied weights parsimony	maximum likelihood	Bayesian
asymmetrical generating phylogeny				
100	34.89 (22–56)	37.85 (22–56)	45.84 (20–58)	28.1 (18–39)
350	26.57 (11–51)	29.2 (12–51)	26.49 (6–58)	19.21 (7–35)
1000	17.82 (3–40)	19.16 (2–33)	11.94 (0–58)	9.34 (0–31)
symmetrical generating phylogeny				
100	8.08 (0–33)	9.29 (0–29)	10.1 (0–58)	7.51 (0–29)
350	1.33 (0–28)	1.43 (0–28)	1.8 (0–52)	1.2 (0–28)
1000	0.32 (0–26)	0.31 (0–26)	0.51 (0–52)	0.31 (0–26)

Nodes closer to the tips are significantly more accurately reconstructed in the asymmetrical phylogenies across all dataset sizes (table 2 and figure 2; electronic supplementary material, figure S8). In the symmetrical trees, there was no significant correlation between distance from the tips and the accuracy of node reconstruction, except in the maximum-likelihood analysis of 100 characters (figure 2 and table 2).

**Table 2.** *p*-Values from Spearman's rank correlation between the percentage of nodes being accurately reconstructed and their distance from the root. Nodes closer to the tips are significantly more likely to be accurately reconstructed in asymmetrical trees but this is not generally true for symmetrical phylogenies.

	asymmetrical tree	symmetrical tree
MB 100	<0.001	0.09919
maximum likelihood 100	<0.001	0.027295
EW 100	<0.001	0.106712
IW 100	<0.001	0.092736
MB 350	<0.001	0.638242
maximum likelihood 350	<0.001	0.057809
EW 350	<0.001	0.19683
IW 350	<0.001	0.148108
MB 1000	<0.001	0.256976
maximum likelihood 1000	<0.001	0.085987
EW 1000	<0.001	0.179186
IW 1000	<0.001	0.287058

### (b) Empirical phylogenies

Patterns of resolution achieved from the simulated datasets are similar for the empirical datasets. The Bayesian implementation of the Mk model estimates the least resolved phylogenies and maximum likelihood produces fully resolved trees (full trees are shown electronic supplementary material, figure S9–S15).

**Figure 4.**
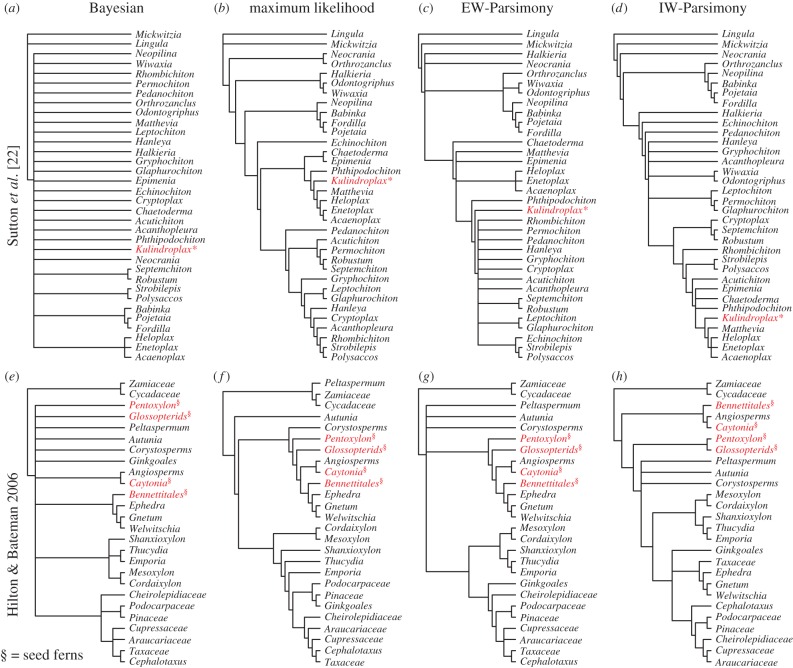
Alternative phylogenetic reconstruction methods alter our understanding of evolution with empirical matrices. However, the relationship of fossil seed ferns from Hilton & Bateman [19] is changed according to implementation (*a–d*), although *Caytonia* remains as sister to angiosperms in all analyses. Alternative analyses change the taxonomic affinity of *Kulindroplax* from Sutton *et al*. [22] (*e–h*). (Online version in colour.)

*Kulindroplax*, from the Sutton *et al.* [22] dataset, is supported as a crown-mollusc based on maximum likelihood, EW-Parsimony and IW-Parsimony (figure 4*a–d*). The results of the IW-Parsimony analysis are most similar to the original results [22], with *Kulindroplax* resolved as a crown-aplacophoran; maximum-likelihood analysis of the dataset resolved *Kulindroplax* as the stem-aplacophoran. The result of the Bayesian analysis of the dataset is largely unresolved, and *Kulindroplax* is not discriminated as a member of any clade within molluscs or even as a member of total-group Mollusca.

The anthophyte hypothesis (non-monophyletic gymnosperms sister to seed ferns plus angiosperms) recovered by Hilton & Bateman [19] is supported by our EW-Parsimony and maximum-likelihood analyses of their dataset which recovered a paraphyletic seed ferns plus Gnetophyta as sister to angiosperms (figure 4*f,g*); the results of Bayesian and IW-Parsimony analyses of the same dataset contradict the anthophyte hypothesis (figure 4*e,h*). The Bayesian analysis produced a non-monophyletic gymnosperms with the relationships between them and seed ferns unresolved with the exception of *Bennettitales* which resolved as a gnetophyte, and *Caytonia* as sister to the angiosperms.

**Figure 5.**
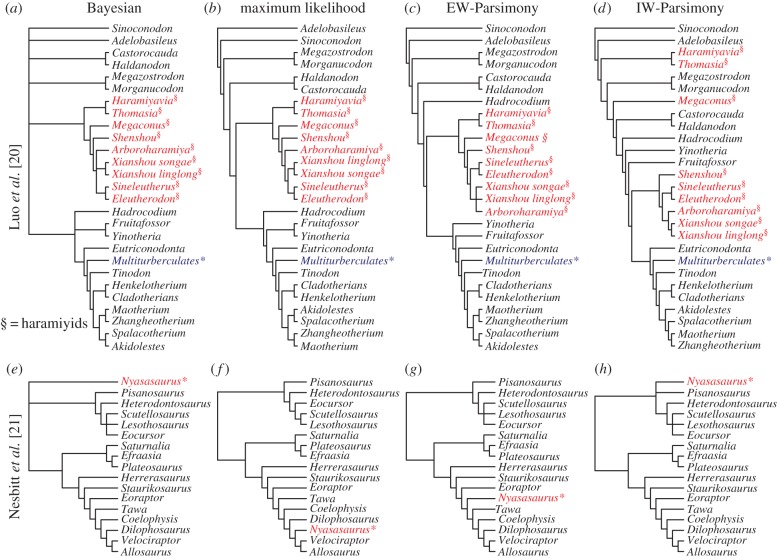
Alternative phylogenetic reconstruction methods produce generally congruent reconstructions of evolution with empirical matrices. For Luo *et al*. [20], the relationship between the haramiyids and multituberculates is largely unchanged across analyses (*a–d*). IW-Parsimony (*g*) and Bayesian analyses place *Nyasasaurus* as close to the earliest dinosaur (*e*) and IW-Parsimony places it close to the earliest diverging taxa (*g*), but EW-Parsimony and maximum likelihood place the taxa as a derived member of Dinosauria (*f,h*). (Online version in colour.)

Analyses of the Luo *et al.* [20] dataset yielded congruent results with the original study, with the placement of *Haramiyavia* outside of crown-Mammalia and multituberculates, although some haramiyids are resolved as crown mammals in the IW-Parsimony analysis (figure 5*a–d*).

*Nyasasaurus* is recovered as a member of Dinosauria in the maximum likelihood, EW-Parsimony and IW-Parsimony analyses of the dataset from Nesbitt *et al.* [21] (figure 5*e–h*). The Bayesian analysis recovers *Nyasasaurus* in a polytomy with the two major clades of dinosaurs, corroborating the conclusion of Nesbitt *et al.* [21] that, given the data, its precise phylogenetic position is uncertain.

## 4. Discussion

### (a) Simulations indicate that the Bayesian implementation of the Mk model outperforms all other methods and implementations

Previous simulation-based analyses that have attempted to evaluate the performance of likelihood and parsimony-based phylogenetic methods for analysing phenotypic data have found that the probabilistic model performs best [7,8]. However, these studies were biased against parsimony because they employed an unresolved generating tree that is problematic as parsimony methods will attempt to recover a fully resolved tree from the simulated data yielding a non-zero RF distance from the generating tree, even if the two trees are effectively compatible. Furthermore, since previous simulation studies considered the Mk model only within a Bayesian framework, they did not distinguish between the impact of the probabilistic model of character evolution and the statistical framework in which it was implemented.

Our analyses control for these shortcomings of previous simulation studies and show consistently that the Bayesian implementation of the Mk model performs best. In line with previous simulations [8], we found that EW-Parsimony performs better than IW-Parsimony. There is overlap between model performance shown by the distribution of Robinson–Foulds distances (table 1), but there is reason to have different degrees of confidence in the models; only the Bayesian implementation produces a relatively small distribution of tree performance compared with the large tails signifying worse performance in the two parsimony methods (table 1). We also found that the Bayesian implementation of the Mk model outperforms the maximum-likelihood implementation, indicating that it is not merely the probabilistic transition model that outperforms parsimony methods, but the implementation of the Mk model within a Bayesian statistical framework. Indeed, the maximum-likelihood implementation of the Mk model was the worst-performing method, worse even than IW-Parsimony. In part, the poor performance of the maximum-likelihood-Mk method is because we did not capture phylogenetic uncertainty associated with this phylogenetic method. This is normally achieved in analyses of molecular datasets through bootstrapping methods, but these are inappropriate for the analysis of phenotypic data as the basic methodological assumption, that the phylogenetic signal is randomly distributed across sites (characters), is not true for morphological data.

However, irrespective of the phylogenetic method used, dataset size correlated positively with both phylogenetic accuracy and resolution, diminishing differences in the relative performance of the competing phylogenetic methods. All phylogenetic methods also performed best when attempting to recover a symmetrical target tree; all methods found recovery of asymmetrical trees challenging and phylogenetic accuracy diminished from tip to root. The impact of tree topology is of particular concern since empirical phylogenetic trees are invariably asymmetric [23], and trees of fossil species are infamous for their asymmetry [24,25]. However, there is a broad spectrum of tree symmetry, with fully symmetric and fully asymmetric trees representing end-members. Palaeontological trees with the dimensions used in our simulations are typically far from the fully asymmetric pectinate-generating tree we employed (Ic = ∼0.4 for 32 species) [25]. Furthermore, the asymmetry of many palaeontological trees is often a representational artefact of attempting to summarize character evolution, or an analytic artefact of analysing the relationships among diverse clades based on representative species or higher taxa [26]. Thus, the challenge of recovering trees of extinct taxa may not be as great as a simplistic interpretation of our results might suggest.

### (b) Analyses of empirical data bear out conclusions based on simulations

Maximum-likelihood, IW-Parsimony and EW-Parsimony methods of the simulated datasets commonly identify a single optimal tree, but the differences between the optimal trees derived from these methods provides no confidence that any one of the inferred topologies is accurate with reference to the placement of a taxon of interest. This view is corroborated by our reanalysis of empirical datasets which recovered poorly resolved trees using the Bayesian implementation of the Mk model, and in a number of instances, indicate that the conclusions drawn in the corresponding original studies are not supported by the data.

In an extreme example, our re-analyses of the dataset published by Sutton *et al.* [22], which attempted to demonstrate a crown-aplacophoran mollusc affinity for *Kulindroplax*, yielded disparate hypotheses of affinity. EW-Parsimony and IW-Parsimony recovered the published result, while maximum likelihood recovered *Kulindroplax* as a stem-aplacophoran, and Bayesian could not discriminate *Kulindroplax* as a total-group mollusc (figure 4*a*). This poor resolution is unlikely to be a result of poor fossil evidence but, rather, the lack of discriminatory power in the small character matrix. Among the analyses of the dataset from Hilton & Bateman [19], we recovered some of the principal competing topologies that have featured in debate over the affinity of seed plants in past decades. However, the Bayesian analysis of the dataset recovered a topology that is largely unresolved in terms of the relationships among key clades. This suggests that the available data are insufficient to discriminate among the competing hypotheses, and this long-standing debate is largely an artefact of the false resolution of parsimony methods.

Bayesian analyses need not overturn the results from previous analyses based on deterministic phylogenetic methods like EW-Parsimony, IW-Parsimony and maximum likelihood. A phylogenetic position for haramiyids, outside crown-Mammalia, is corroborated by our Bayesian analysis of the dataset from Luo *et al.* [20]—in contrast with the crown-Mammalia affinity recovered for some haramiyids through IW-Parsimony analysis of the same data (figure 5*d*). Similarly, *Nyasasaurus* was posited as the earliest dinosaur, and this conclusion is supported by the Bayesian analyses (figure 5*e*) although this is not supported by EW-Parsimony, IW-Parsimony and maximum-likelihood analyses (figure 5*f–h*). However, the Bayesian analysis is more robust in expressing the phylogenetic ambiguity identified by the original authors [19], as *Nyasasaurus* falls in a polytomy alongside the two major clades of dinosaurs.

Some of the differences between methods may simply reflect the dimensions of the dataset. The two datasets that cannot resolve relationships under Bayesian inference and exhibit significant topological discordance among phylogenetic methods [19,22] are both comparatively small (34 taxa, 48 characters and 48 taxa, 82 characters). These both fall within the scope of simulated datasets that yield low resolution from the Bayesian method and, from other phylogenetic methods, high resolution but low accuracy (figure 1). The two empirical datasets that yield trees with greater congruence from the different phylogenetic methods, are both larger: Luo (114 taxa, 497 characters) and Nesbitt (82 taxa, 413 characters). The size of these matrices is comparable with our simulation results in which we see marked increases in topological accuracy and agreement between methods (figure 1, between 350 and 1000 characters).

### (c) Implications for phylogenetic analysis of phenotypic data

The results of our simulation studies indicate that the cadre of phylogenetic hypotheses generated from phenotypic data using parsimony methods require reassessment using the Bayesian implementation of the Mk model. It is likely that many evolutionary interpretations are contingent on precise but inaccurate phylogenetic hypotheses. In this undertaking, it is important that the implications of our simulation studies are considered in the design of phylogenetic studies.

Firstly, phylogenies of fossils tend towards strong asymmetries [25] and, like all phylogenetic methods, Bayesian inference struggles with the recovery of deep nodes within asymmetric trees. Therefore, it is important that outgroups are sampled extensively, ensuring that contentious in-group relationships are closer to the tips, where topological accuracy is highest. Further, in-group lineages should be sampled in a manner that does not accentuate tree asymmetry.

Secondly, phylogenetic accuracy and resolution correlates positively with the relative dimensions of the dataset. Accordingly, phylogenetic resolution or certainty should not be expected from cladistic analyses of small morphological datasets (i.e. those around 100 characters or fewer), particularly if they include fossils. There are finite limits to the number of available phylogenetically informative characters [27] and, for well-studied clades, it may be perceived that these phylogenetically informative characters have already been found. However, it is important to note that the concept of phylogenetic informativeness is different within a likelihood versus a parsimony framework: in parsimony characters that undergo few changes are prized in favour of homoplastic characters. Under the likelihood model, branch length, informed by the number of character changes, contributes to topology estimation. Thus, traditionally ‘bad’ phylogenetic characters (those exhibiting homoplasy) may find utility in expanding the dimensions of phenotypic character matrices as long as homoplasy falls within the limits that the model can accommodate. In a Bayesian framework, this can be tested using posterior predictive tests of model adequacy (e.g. [28]).

Finally, we may need to alter our expectations to anticipate less well-resolved but more accurate phylogenetic hypotheses, which will both constrain and guide research. Greater resolution may be found by generating matrices suited to likelihood- rather than parsimony-based phylogenetic methods. However, we must also come to terms with the prospect that for some groups of organisms, or their fossil remains, there may be insufficient data. As such, their evolutionary relationships might not therefore be resolvable using morphological data alone and, if they are fossils, their evolutionary significance may never be realized. Nevertheless, resolving phylogenies is not the end game for evolutionary biology. Incompletely resolved trees can still be used as a basis for investigating interesting macroevolutionary questions, and methods exist for incorporating tree uncertainty in phylogenetic comparative methods (e.g. [29]).

## 5. Conclusion

A growing consensus shows that the Bayesian Mk model is the most accurate method of phylogenetic reconstruction, and here we show that this remains true across dramatically different tree shapes, when analysing datasets composed of both multistate and binary characters, and when compared with maximum-likelihood estimation using the Mk model. We recommend that Bayesian implementations of the Mk model should become the default method for phylogenetic analyses of cladistic morphological datasets, and we should expect low levels of resolution with small datasets. As parsimony methods appear to be less effective than probabilistic approaches, it may be necessary to alter data collection practices by moving away from choosing a selection of characters that undergo few changes, and moving towards scoring all possible characters from the available taxa irrespective of their expected homoplasy.

## Supplementary Material

Puttick_et_al_Supplementary_Figures

## Supplementary Material

Puttick_et_al_R_script

## Data accessibility

Supplementary figures and the code used to simulate the data used in this publication can be accessed in the electronic supplementary material.
