# Supplementary material for: Uncertain-tree: discriminating among competing approaches to the phylogenetic analysis of phenotype data
Source: Proc Biol Sci. 2017 Jan 11;284(1846):20162290. doi: 10.1098/rspb.2016.2290 (PMC5247500; doi:10.1098/rspb.2016.2290)
Supplement: Puttick_et_al_Supplementary_Figures [file rspb20162290supp1.docx]

**Supplementary Figure**

**Supplementary Figure S1.** The two phylogenies used for simulations

**Supplementary Figure S2.** Target distribution of CI values for datasets produced from the simulation phylogenies.

**Supplementary Figure S3.** The percentage of nodes resolved from asymmetrical generated trees for all methods and dataset sizes.

**Supplementary Figure S4.** The percentage of nodes resolved from symmetrical generated trees for all methods and dataset sizes.

**Supplementary Figure S5.** Robinson-Foulds scores and for all phylogenies based on the 100 character simulated datasets.

**Supplementary Figure S6.** Robinson-Foulds scores and for all phylogenies for the 350 character simulated datasets.

**Supplementary Figure S7.** Robinson-Foulds scores and for all phylogenies for the 1000 character simulated datasets.

**Supplementary Figure S8.** The accuracy of reconstructions of node against the distance of each node from the root of the phylogeny. For the symmetrical simulation trees (a) there is generally high performance of all nodes. However, the asymmetrical phylogenies show a positive relationship as nodes further from the root are reconstructed more accurately (b).

**Supplementary Figure S9.** Full phylogenies of Sutton et al. (2015) using equal weights parsimony, implied weights parsimony, maximum likelihood and Bayesian.

**Supplementary Figure S10.** Full phylogeny of Luo et al. (2015) using equal weights parsimony.

**Supplementary Figure S11.** Full phylogeny of Luo et al. (2015) using implied weights parsimony.

**Supplementary Figure S12.** Full phylogeny of Luo et al. (2015) using maximum likelihood.

**Supplementary Figure S13.** Full phylogeny of Luo et al. (2015) using Bayesian implementation in MrBayes.

**Supplementary Figure S14.** Full phylogenies of Nesbitt et al. (2013) using equal weights parsimony, implied weights parsimony, maximum likelihood and Bayesian.

**Supplementary Figure S15.** Full phylogenies of Hilton and Bateman (2006) using equal weights parsimony, implied weights parsimony, maximum likelihood and Bayesian.
